# Role of the p53/p21 system in the response of human colon carcinoma cells to Doxorubicin

**DOI:** 10.1186/1471-2407-4-92

**Published:** 2004-12-15

**Authors:** Raffaella Ravizza, Marzia B Gariboldi, Laura Passarelli, Elena Monti

**Affiliations:** 1 Dept. of Structural and Functional Biology, Section of Pharmacology, University of Insubria, Via A. da Giussano 10, 21052 Busto Arsizio (VA), Italy

## Abstract

**Background:**

Colon adenocarcinomas are refractory to a number of widely used anticancer agents. Multifactorial mechanisms have been implicated in this intrinsically resistant phenotype, including deregulation of cell death pathways. In this regard, the p53 protein has a well established role in the control of tumor cell response to DNA damaging agents; however, the relationship between p53-driven genes and drug sensitivity remains controversial. The present study investigates the role of the p53/p21 system in the response of human colon carcinoma cells to treatment with the cytotoxic agent doxorubicin (DOX) and the possibility to modify the therapeutic index of DOX by modulation of p53 and/or p21 protein levels.

**Methods:**

The relationship between p53 and p21 protein levels and the cytotoxic effect of DOX was investigated, by MTT assay and western blot analysis, in HCT116 (p53-positive) and HT29 (p53-negative) colon cancer cells. We then assessed the effects of DOX in two isogenic cell lines derived from HCT116 by abrogating the expression and/or function of p53 and p21 (HCT116-E6 and HCT116 p21-/-, respectively). Finally, we evaluated the effect of pre-treatment with the piperidine nitroxide Tempol (TPL), an agent that was reported to induce p21 expression irrespective of p53 status, on the cytotoxicity of DOX in the four cell lines. Comparisons of IC50 values and apoptotic cell percentages were performed by ANOVA and Bonferroni's test for independent samples. C.I. calculations were performed by the combination Index method.

**Results:**

Our results indicate that, in the colon carcinoma cell lines tested, sensitivity to DOX is associated with p21 upregulation upon drug exposure, and DOX cytotoxicity is potentiated by pre-treatment with TPL, but only in those cell lines in which p21 can be upregulated.

**Conclusions:**

p21 induction may significantly contribute to the response of colon adenocarcinomas cells to DOX treatment; and small molecules that can exploit p53-independent pathways for p21 induction, such as TPL, may find a place in chemotherapeutic protocols for the clinical management of colorectal cancer, where p53 function is often lost, due to genetic or epigenetic defects or to post-transcriptional inactivating mechanisms.

## Background

Colorectal cancer is the second most common cause of cancer-related mortality in Western countries, with about 1 million new cases every year diagnosed world-wide and 500,000 patients dying from the disease [1]. Of the patients, 30% have advanced disease at presentation, either locally or at distant sites; in this setting, chemotherapy remains the only viable therapeutic option. However, even this option is severely hindered by the inherent resistance of metastatic colon cancer to many currently used anticancer agents. A variety of mechanisms by which cancer cells resist chemotherapy have been described, including enhanced export of drugs from cancer cells and alterations in drug metabolism and/or in drug-target interactions [2]. In addition, the response of cancer cells to genotoxic therapies may be critically impaired by defects in the response mechanisms to DNA damage [3] or in cell cycle regulatory pathways [4].

Over the past decade, induction of apoptosis has emerged as a major event in tumor cell response to cytotoxic agents (for a recent review see [5]). This view, although recently challenged by some Authors [6], has attracted considerable attention on deregulation of cell death pathways as a key determinant of drug resistance.

Two separable, although extensively cross-talking, pathways leading to apoptosis have been characterized [7,8]. The *extrinsic pathway *is initiated by ligation of transmembrane receptors to activate membrane proximal "activator" caspases, which in turn cleave and activate downstream "effector" caspases. The *intrinsic pathway *requires disruption of the mitochondrial membrane and the release of mitochondrial proteins, two events that are regulated by the opposing actions of pro- and antiapoptotic Bcl-2 family members. "Intrinsic stresses", such as those produced by DNA-damaging agents, activate the intrinsic apoptotic pathway; the multifunctional transcription factor p53 is thought to be part of a "fast track" connection between nuclear DNA damage and the intrinsic pathway machinery [9]. *p53 *regulates multiple responses to genotoxic stress by transcriptional activation or repression of a number of genes encoding proteins involved in cell cycle control (p21^WAF1/Cip1^), DNA repair (gadd45), and apoptosis (e.g. Bax, Bcl2 and survivin) [10]. Mutations in *p53 *and in the p53 pathway can produce multidrug resistance *in vitro *and *in vivo*, and reintroduction of wildtype *p53 *into *p53 *null tumor cells can re-establish chemosensitivity [11]. *p53 *status is not a universal predictor of treatment response, in part because not all drugs absolutely require p53 for their apoptotic function [12] and in some settings, p53 loss can enhance drug-induced apoptotic cell death [13]. Still, loss of p53 function correlates with multidrug resistance in many tumor types [11] and the observation that this is a common defect in human tumors has spurred an active search for strategies aimed at directly activating cell death pathways downstream of p53. In this scenario, the role played by the cyclin-dependent kinase inhibitor p21 is particularly intriguing, as this protein can be activated by both p53-dependent and p53-independent mechanisms and can assume pro- or anti-apoptotic functions, depending on the cellular context (for a review see [14]).

**Figure 1:**
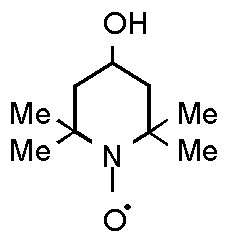
Structure of 4-hydroxy-2,2,6,6,tetramethylpiperidne-*N*-oxyl (also known as Tempol, TPL)

The present research focuses on the role of p21 in tumor cell response to treatment with cytotoxic agents, and on the possibility to improve the therapeutic index of such agents by modulating p21 status by p53-dependent and independent pathways. The following issues have been addressed: (a) analysis of the relationship between p21 status and sensitivity to treatment with the cytotoxic anticancer agent doxorubicin (DOX) in p53-positive and -negative colon cancer cell lines; (b) design of treatment strategies based on the use of small molecules able to modulate p21 status; for the present study we have used a low molecular weight, stable nitroxide radical, 4-hydroxy-2,2,6,6,tetramethylpiperidne-*N*-oxyl (also known as Tempol, TPL; figure 1) that was shown to exert an antiproliferative effect against different cancer cell lines [15] and to increase p21 levels in a p53-null human leukemic cell line [16]. Our data indicate that p21 modulation may significantly affect cell response to DOX treatment in the colon cancer cell lines tested.

## Methods

### Reagents

Standard chemicals, including 4-hydroxy-2,2,6,6-tetramethylipiperidine-*N*-oxyl (Tempol, TPL) and cell culture reagents were purchased from Sigma-Aldrich srl. (Milan, Italy), unless otherwise indicated; doxorubicin (DOX) was kindly provided by Dr. A Suarato (Pfizer-Pharmacia, Milan, Italy).

### Cell lines

The human colon carcinoma cell lines HCT116, HT29 (obtained from ATCC, Rockville, MD) and HCT116 p21-negative cells (HCT116 p21-/-), kindly provided by Dr B. Vogelstein (Johns Hopkins University, Baltimore, MD, USA), were maintained in DMEM medium supplemented with 10% FBS (Mascia Brunelli); the HCT116-E6 cell line, obtained from HCT116 cells by transfection with pCMVneo-E6 plasmid (provided by Dr B. Vogelstein) containing the HPV16-E6 human gene, was maintained in ISCOVE medium supplemented with 10% FBS and geneticin (500 μg/ml). All the cell lines were cultured at 37°C, in an atmosphere of 5% CO_2 _and 95% humidity.

### Cytotoxicity assays

The effects of DOX and/or TPL on cell growth were assessed by the MTT assay [17]. Briefly, cells were seeded onto 96-well plates and allowed to grow for 24 h prior to treatment. Three different treatment schedules were used: a) 24 h medium, 1 h DOX (0.05 – 10 μM) followed by 72 h incubation in drug-free medium; b) 24 h TPL (0.05 – 10 mM) followed by 72 h incubation in drug-free medium; c) 24 h TPL followed by 1 h DOX and by 72 h incubation in drug-free medium. For combination experiments, the whole range of DOX concentrations (0.05 – 10 μM) was tested following pretreatment with fixed TPL concentrations, corresponding to the IC_25 _or IC_50_values obtained for each cell line according to schedule (b). At the end of the treatment period, 50 μl of MTT (2 mg/ml in PBS) were added to each well at 37°C for 3 h and the reduction of MTT by viable cells was measured colorimetrically at 570 nm, using a Universal Microplate Reader EL800 (Bio-Tek Instruments). IC_25 _and IC_50 _values (i.e. the concentrations yielding 75% and 50% cell survival fractions, respectively) were calculated according to the median effect equation and analysis of the interaction between DOX and TPL was performed as described by Chou & Talalay [18].

### Transfection of HCT116 cells

Transfection of HCT116 cells with the pCMVneo-E6 plasmid was performed by electroporation as described by Yanez and Porter [19], using a Bio-Rad Gene Pulser unit at the following conditions: 280 V, 960 μF. 48 h post-transfection the cells were selected by adding 500 μg/ml of geneticin to the culture medium. The efficiency and stability of transfection were checked by Western blot analysis of whole cell lysates. Control cells were mock-transfected with the pCMVneo plasmid.

### Preparation of cell extracts and immunoblotting

The expression of p53 and p21, before and after 1 h exposure to DOX (1.0 and 10 μM) followed by 23 h incubation in drug-free medium, or after 24 h exposure to TPL (1.0 and 2.5 mM), was evaluated by Western blot analysis of total protein extracts (lysis buffer: NP40 1%, leupeptin 10 μg/ml and aprotinin 10 μg/ml in TBS). Protein concentration in the cellular lysates was determined by the BCA assay (Pierce, Rockford, IL, USA). 15 μg of protein extract/lane were loaded onto 11% polyacrylamide gels and separated under denaturing conditions. Protein samples were then transferred onto nitrocellulose membrane and Western blot analysis was performed by standard techniques. using a mouse anti-p53 monoclonal antibody (DO-1; Santa Cruz Biotechnology, Inc., Santa Cruz, CA, USA) and a rabbit anti-p21 polyclonal antibody (C-19; Santa Cruz Biotechnology). Proteins were visualized using peroxidase-conjugated anti-mouse and anti-rabbit secondary antibodies (Amersham Pharmacia Biotech) and the ECL Plus Western Blotting Detection Reagents (Amersham Pharmacia Biotech).

Densitometric analysis was performed using the Scion Image software (Scion Corporation, Frederick, MD).

### Flow cytometric analysis of apoptotic cells

The presence of apoptotic cells in HCT116, HT29, HCT116-E6 and HCT116 p21 -/-, before and after 1 h exposure to DOX (1.0 and 10 μM) followed by 23 h incubation in drug-free medium, was evaluated by flow cytometric analysis, using a Becton Dickinson FACScalibur flow cytometer. Cells were detached by trypsinization, washed in phosphate-buffered saline (PBS) and fixed in ice-cold 70% ethanol for 20 min at -20°C. After an additional wash in PBS, DNA was stained with 50 mg/ml propidium iodide in PBS in the presence of RNAse A (30 U/ml) at 37°C for 30 minutes. 5 × 10^5 ^cell samples were analyzed and data were processed using the CellQuest software (Becton Dickinson). The percentage of apoptotic cells in each sample was determined based on the sub-G1 peaks detected in monoparametric histograms.

### Statistical analysis

Comparisons of IC_50 _values and apoptotic cell percentages in the four different cell lines were performed by ANOVA and Bonferroni's test for independent samples. C.I. calculations an relative statistical analysis were performed as described by Chou and Talalay [18]. According to this method, a *combination index *(C.I.) can be calculated from dose-response curves obtained following exposure to DOX and/or TPL as single agents and in combination. C.I. values approximating 1.0 indicate additive interactions between the two agents; C.I. < 1.0 indicate synergy and, conversely, C.I. > 1.0 indicate antagonism.

## Results

### Cytotoxicity assays

**Figure 2:**
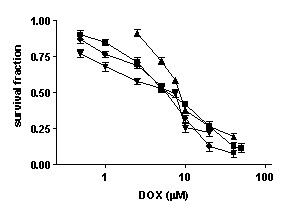
Dose-response curves of HCT116 (■), HT29 (▲), HCT116E6 (▼), and HCT116 p21-/- (●) cells after 1 hour exposure to DOX followed by 72 h incubation in drug free medium (mean ± s.e.m. of 4–6 experiments).

Figure 2 shows the dose-response curves for HCT116, HT29, HCT116-E6 and HCT116 p21-/- cells following 24 h incubation in drug-free medium, 1 h exposure to DOX and 72 h incubation in drug-free medium. IC_50 _values calculated from these data are reported in table 1; the *resistance index *(R.I.) is calculated as the ratio between the IC_50 _value obtained for each cell line and that obtained for HCT116 cells. The HT29 cell line, which carries a mutant form of the *p53 *gene, is significantly more resistant to the cytotoxic action of DOX than the HCT116 cell line, carrying a wild-type *p53 *gene (IC_50 _values: 2.197 ± 0.11 μM vs. 0.38 ± 0.03 μM, respectively; mean ± s.e.m. of 4–6 experiments, *p *< 0.05). HCT116-E6 cells are 2-fold more resistant to DOX than HCT116 cells (IC_50_: 0.770 ± 0.06 μM; mean ± s.e.m. of 4–6 experiments, *p *< 0.05 vs. HCT116), while HCT116 p21-/- cells are 14-fold more resistant to DOX than HCT116 cell line (IC_50_: 5.457 ± 0.163 μM; mean ± s.e.m. of 4–6 experiments, *p *< 0.05 vs. HCT116), in spite of the presence of a wild type form of p53.

### Immune detection of p53 and p21

**Figure 3:**
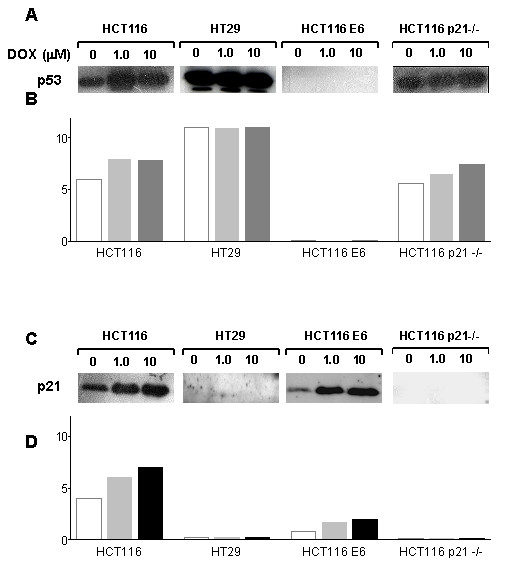
p53 (A) and p21 (C) protein levels in HCT116, HT29, HCT116 E6 and HCT116 p21-/- cells following 1 h exposure to DOX and 23 h incubation in drug-free medium. Panels B and D: densitometric analysis of p53 and p21-immune reactive bands, respectively (white bars: untreated; light grey bars: DOX 1 μM; dark grey bars: DOX 10 μM).

Figure 3 shows the expression of p53 (A) and p21 (C) proteins, before and after exposure to DOX (1 and 10 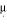
M for 1 h followed by 23 h in drug-free medium), in the four cell lines tested; panels B and D report the densitometric analysis of p53 and p21 immune reactive bands, respectively. In the absence of drug treatment, it is possible to observe that HT29 cells show higher p53 but lower p21 protein levels, as compared with the HCT116 cell line. DOX treatment induces a dose-dependent increase in both p53 and p21 levels in HCT116 cells, whereas in HT29 cells p53 protein levels are not significantly modified by the treatment and p21 protein levels are only detectable when 25 μg of protein extract/lane are loaded onto the gel (instead of the 15 μg loaded for the other cell lines), and even then only at the highest DOX concentration used. As expected, HCT116 E6 cells do not show detectable p53 levels, both under baseline conditions and following DOX treatment; in contrast in HCT116-E6 cells p21 levels are increased by DOX treatment in a dose-dependent fashion, although to a lesser extent than in HCT116 cells. HCT116 p21-/- cells show higher baseline expression levels of p53 compared with HCT116 cells and DOX treatment in this cell line enhances p53 expression to a an even greater extent than in HCT116 cells line. As expected, p21 is undetectable in HCT116 p21-/- cells, and DOX treatment does not modify the intracellular levels of this protein.

### Evaluation of apoptotic cells by flow cytometric analysis

**Figure 4:**
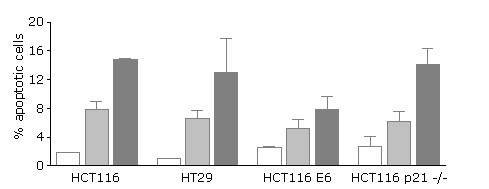
Percentage of apoptotic cells in HCT116, HT29, HCT116 E6 and HCT116 p21-/- cells before and after 1 h exposure to DOX followed by 23 h incubation in drug-free medium (white bars: untreated; light grey bars: DOX 1 μM; dark grey bars: DOX 10 μM). Mean ± s.e.m. of 3 independent experiments.

Figure 4 shows the percentage of apoptotic cells following treatment of the four colon cell lines with DOX (1.0 and 10 μM) for 1 h followed by 23 h in drug-free medium. No significant differences in the percentage of apoptotic cells were observed in untreated HCT116, HT29, HCT116-E6 and HCT116 p21-/- cells. Exposure to DOX induces concentration-dependent increases in apoptotic cells in all the cell lines tested; HCT116-E6 cells were the least susceptible apoptosis induction by DOX.

### Effects of TPL on cell survival and p53/p21 levels

**Figure 5:**
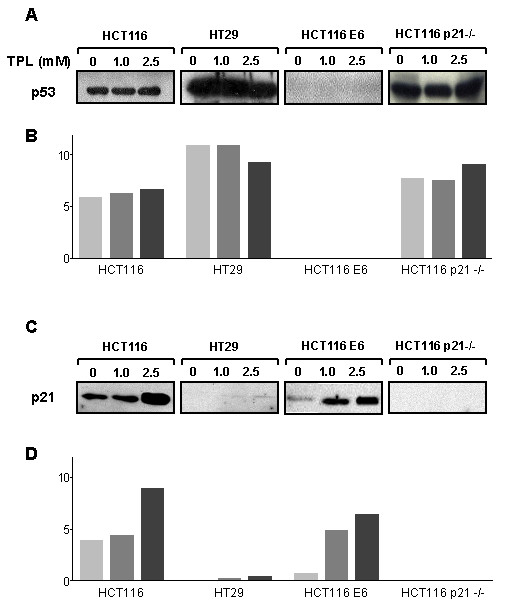
p53 (A) and p21 (C) protein levels in HCT116, HT29, HCT116 E6 and HCT116 p21-/- cells following 24 h exposure to TPL. Panels B and D: densitometric analysis of p53 and p21-immune reactive bands, respectively (white bars: untreated; light grey bars: TPL 1.0 mM; dark grey bars: TPL 2.5 mM).

Table 2 reports the IC_25 _and IC_50 _values obtained for the four cell lines after 24 h of continuous TPL exposure followed by 72 h in drug-free medium. TPL can be observed to inhibit cell growth in all four cell lines; although no significant differences can be detected among IC_50 _values, HCT116 p21-/- cells appear to be less responsive than the other three cell lines. Figure 5 shows that 24 h exposure to TPL (1 and 2.5 mM) induces a dose-dependent increase in both p53 and in p21 levels in HCT116 cells, whereas in HT29 cell line TPL treatment only induces a dose-dependent increase in p21 expression. Exposure of HCT116-E6 cells to TPL (1 and 2.5 mM) for 24 h does not induce any variations in p53 expression, while a dose-dependent increase in p21 expression can be observed following treatment with the nitroxide. In HCT116 p21-/- cells TPL induce a slight increase in p53 protein levels but, as expected, p21 levels were unaffected by TPL treatment.

### Effects of TPL pretreatment on DOX-induced cytotoxicity

**Figure 6:**
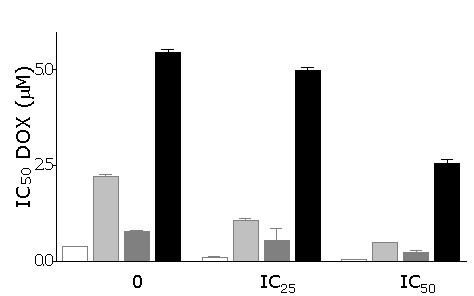
Effect of 24 h pre-treatment with TPL on DOX IC_50 _values obtained in HCT116, HT29, HCT116 E6 and HCT116 p21-/- cells following 1 h exposure to DOX and 72 h in drug-free medium. For each cell line, two TPL concentrations corresponding to IC_25 _and IC_50 _(see table 2), were used. Mean ± s.e.m. of 4–6 experiments.

Figure 6 shows the effect of 24 h pretreatment with TPL, at fixed concentrations corresponding to the IC_25 _and IC_50 _values obtained for each cell line, on DOX cytotoxicity. The cells' response to DOX is expressed as the IC_50 _values derived from dose/response curves obtained after 1 h exposure to DOX with or without pretreatment with TPL (24 h), followed by 72 h in drug-free medium. Analysis of cytotoxicity data shows a synergistic interaction (C.I.<1) between DOX and TPL for both TPL concentrations in HCT116 cells and in HCT116-E6 and HT29 cells at the lower concentration; only additive effects (C.I. ≈ 1) can be observed in HCT116 p21 -/- cells. IC_50 _values for DOX and TPL according to the three different schedules are reported in table 3.

## Discussion

Resistance of colorectal cancer to established treatment regimens remains a major concern in oncology; thus attempts at improving the survival of patients affected by this disease depend largely on strategies targeting tumor cell resistance, which cannot be rationally planned without a detailed knowledge of the mechanisms underlying this phenomenon. A current paradigm regarding cancer chemotherapy indicates disabling of the intrinsic apoptotic pathway as a key factor in the response of tumor cells to anticancer drugs [3,5,12]. Therefore, strategies aiming at re-establishing the cell's capability to activate a cell death program are an area of active research. The present study was performed in order to define the role of the p53/p21 pathway in the response of colorectal carcinoma cells to DOX, a cytotoxic agent that is typically devoid of effects in this tumor type. The results obtained in our cytotoxicity studies indicate that in the cell lines examined *p53 *status is not unequivocally related to the response to DOX: in fact, while *p53*-deficient cells (HT-29, HCT116-E6) are indeed less responsive than the *p53/wt *parental HCT116 cell line, the highest resistance index was obtained for HCT116 p21-/- cells, harboring two wildtype *p53 *alleles. As expected, treatment with DOX leads to p53 upregulation in the cell lines expressing wildtype p53; this effect has been thoroughly documented in colon cancer cells as well as in tumor cell lines derived from other tissues, and has been attributed to phosphorylation and subsequent stabilization of p53, possibly through activation of DNA-dependent protein kinase or ATM (ataxia-teleangectasia mutated) kinase (see e.g. [20-22]). In HCT116 cells, p21 expression parallels p53 activation; however, data obtained in HT29 and HCT116-E6 cells clearly indicate the existence of p53-independent pathways for p21 induction, that have been extensively characterized (for a review see [23]) and can be activated to variable extents (HCT116 E6 > HT29) upon exposure to DOX. Interestingly, the extent of the cytotoxic effects observed in the small panel of colon cell lines tested rather seems to parallel the cells' ability to upregulate p21 (HCT116 > HCT116-E6 > HT29 > HCT116 p21-/-). This result is somewhat unexpected: in fact, whereas the function of p21 in cell growth arrest following DNA damage has been established for a long time [24], the role played by this protein in the ultimate fate of tumor cells exposed to cytotoxic agents is far from clear-cut [14,25]. In a number of studies, p21 has actually been reported to protect tumor cells against cell death induced by enforced p53 expression [26] or by low doses of cytotoxic agents [13,27-30]. However, in other experimental settings, p53-dependent or -independent induction of p21 expression seems to be a prerequisite for apoptosis [31-34] and to sensitize tumor cells to the action of different agents [35-37]. The putative mechanisms by which p21 might actually induce apoptosis have recently been reviewed [38], but still await full elucidation.

Interestingly, the situation outlined by our results does not seem to conform to either view: in fact, while induction of p21 in p53-proficient and -deficient cell lines is associated with increased response to drug treatment, this was not accompanied by a parallel increase in apoptotic cells, as no significant differences in apoptosis were observed between HCT116 cells and the 14-fold resistant HCT-116 p21-/- cell line (figure 4). This suggests that modes of cell death other than apoptosis may operate in tumor cells following exposure to DOX, or, more generally, to DNA-damaging agents, a concept that is beginning to be proposed by a number of Authors [39,40]; of note, recent experimental evidence indicates p21 as one of the major determinants of terminal growth arrest induced by cytotoxic agents [41-43]. Therefore, although issues related to terminal growth arrest and senescence have not been specifically addressed in the present study, the possibility that these phenomena might play a role linking cell death to the observed increases in p21 levels should not be disregarded.

The hypothesis that the cytotoxic response of the tumor cell lines tested in the present study may depend on p21 induction is further corroborated by data obtained following pre-treatment with the piperidine nitroxide TPL. The choice of this compound was dictated by previous findings indicating that TPL induces cell death in a number of tumor cell lines irrespective of their *p53 *status [15], and that it increases p21 levels in *p53*-null cells [16]. The results of the present study show that TPL affects the four colon cell lines to similar extents, thus confirming that its growth inhibitory effect is independent of p53 function. HCT-116 p21-/- cells are actually slightly less responsive than the other cell lines [even though the difference does not attain statistical significance), which suggests the possibility that the effects of TPL are due in part to its ability to increase p21 levels. Interestingly, the nitroxide also induces p21 expression even in p53-deficient cell lines; this observation suggests that TPL can activate p53-independent pathways for p21 induction, as already noted following exposure of HT29 and HCT116 E6 cells to DOX. Moreover, activation of such pathways by TPL appears to sensitize tumor cells to the action of DOX: in fact, synergistic potentiation of DOX cytotoxicity is achieved by TPL in those cell lines where p21 expression can be induced, but only additive effects between TPL and DOX are observed in HCT116 p21-/-, where p21 expression is constitutively absent.

## Conclusions

In summary, the results of the present study strongly suggest that 1) p21 induction may significantly contribute to the response of colon adenocarcinoma cells to DOX treatment; and 2) small molecules that can exploit p53-independent pathways for p21 induction, such as TPL, may find a place in chemotherapeutic protocols for the clinical management of colorectal cancer, where p53 function is often lost, due to genetic or epigenetic defects or to post-transcriptional inactivating mechanisms.

## Competing interests

The author(s) declare that they have no competing interests

## Pre-publication history

The pre-publication history for this paper can be accessed here:



## Figures and Tables

**Table 1:** IC_50_values obtained after 1 h exposure to DOX followed by 72 h incubation in drug free medium (mean ± s.e.m. of 4–6 independent experiments).

cell lines	IC_50 _(μM)	R.I.^a^
HCT116	0.38 ± 0.03	1.0
HT29	2.37 ± 0.13 *	6.24
HCT116 E6	0.732 ± 0.06 *	1.93
HCT116 p21-/-	4.98 ± 0.32 *	13.10

*p < 0.05 vs HCT116

^a^The *resistance index *(R.I.) was calculated as the ratio between the IC_50 _values obtained for each cell line and that of HCT116 cells

**Table 2:** IC_50_values obtained after 24 h exposure to TPL followed by 72 h incubation in drug free medium. (mean ± s.e.m. of 4–6 independent experiments).

Cell lines	IC_25 _(μM)	IC_50 _(μM)	R.I.^a^
HCT116	280.00 ± 39.21	560.00 ± 77.78	1.0
HT29	320.40 ± 50.03	665.63 ± 102.91	1.19
HCT116 E6	400.06 ± 60.00	837.3 ± 103.99	1.49
HCT116 p21-/-	700.00 ± 99.14	1114.55 ± 182.2	1.99

^a ^The *resistance index *(R.I.) was calculated as the ratio between the IC_50 _values obtained for each cell line and that of HCT116 cells

**Table 3:** IC_50_values obtained after 24 h exposure to TPL followed by 1 h exposure to DOX and 72 h incubation in drug free medium (mean ± s.e.m. of 4–6 independent experiments).

	DOX	+ TPL (IC_25_)	+ TPL (IC_50_)	C.I.^a^
HCT116	0.38 ± 0.03	0.102 ± 0.04	0.053 ± 0.003	0.4–0.6
HT29	2.20 ± 0.11	1.06 ± 0.12	0.49 ± 0.015	0.6–0.8
HCT116 E6	0.77 ± 0.06	0.54 ± 0.6	0.24 ± 0.07	0.6–0.8
HCT116 p21-/-	5.457 ± 0.16	4.98 ± 0.15	2.549 ± 0.21	0.8–1.1

^a^*Combination index *(C.I.) ≈ 1.0: additivity; C.I.<1.0: synergy; C.I.>1.0: antagonism
